# Misdiagnosis of persistent asthma of a patient suffering from acute bronchopulmonary aspergillosis (ABPA)

**DOI:** 10.1016/j.amsu.2021.102696

**Published:** 2021-08-09

**Authors:** Yousef S. Abuzneid, Yasmine Yaghi, Arein Madia, Nataly Salhab, Naser Amro, Sadi A. Abukhalaf, Mohammad Kharraz

**Affiliations:** a Al-Quds University Faculty of Medicine, Jerusalem, Palestine; b An-Najah National University Hospital, Nablus, Palestine

**Keywords:** Acute bronchopulmonary aspergillosis, Asthma, Immune response, Case report

## Abstract

**Background:**

Allergic bronchopulmonary aspergillosis (ABPA) is a condition characterized by an exaggerated immune response (a hypersensitivity response) to the fungus *Aspergillus* (most commonly *Aspergillus fumigatus*).

ABPA causes airway inflammation that if left untreated can lead to bronchiectasis (an abnormal dilation of the airways) due to the immune system and fungal spores damaging sensitive lung tissues and ultimately leading to scarring.

**Case presentation:**

We present a case of a 32-year-old female patient who was misdiagnosed with persistent asthma and treated accordingly for several months until a reexamination was done and a diagnosis of ABPA was concluded. Treatment was altered which led to the successful recovery of the patient.

**Conclusion:**

A reevaluation of the patients’ condition was needed to arrive to the correct diagnosis and to put her on the correct treatment as an ABPA patient instead of persistent asthma, concluding that the medical history and physical examination are both of vital significance to stipulate a correct diagnosis.

## Highlights

• Acute bronchopulmonary aspergillosis can be misdiagnosed for other conditions such cystic fibrosis or asthma.
• Careful history taking, meticulous physical examination, and investigations are necessary.
• Prompt treatment should be address in a proper way to kill the fungus.

## 1 Introduction

Allergic bronchopulmonary aspergillosis is an airway hypersensitivity reaction to the fungus *A. fumigatus* antigens that is almost seen exclusively in patients with asthma and cystic fibrosis. Only a minority of the population develop this condition after the aspergillus hypersensitivity (which is defined as the presence of cutaneous hypersensitivity to the above-mentioned fungus) [1,3].

The prevalence of this disease among patients with asthma is approximately 1–2% but slightly higher in patients that suffer from cystic fibrosis being about 2–9% [1,4]. For those that are corticosteroids-dependent asthmatics, the prevalence is 7–14% [11].

There are no gender predilections noted and the global burden for this disease potentially exceeds 4.8 million people [2,4].

This disease can also be seen in recipients of a lung transplant and people that suffer from bronchiectasis, chronic granulomatous disease or hyper IgE syndrome, but it is very rare among those individuals [3].

There are debates about the predisposing factors that can lead to ABPA, but they are thought to include atopy, immunogenic HLA-restricted phenotypes, mutations in the CFTR gene that causes cystic fibrosis, polymorphisms of the collagen region of the surfactant protein A2, psychochemical characteristics of respiratory secretions and environmental exposure history [5].

The work has been reported in line with the SCARE 2020 criteria [13].

## 2 Case presentation

A 32-year-old female patient with a history of bronchial asthma of two-year duration came to our hospital for evaluation of uncontrolled asthma. Since her diagnosis, she was started on short acting β-agonists followed by inhaled steroids.

Not long after, long acting β-agonists and Montelukast were added to the treatment because of persistent asthma consisting of a persistent cough, shortness of breath and wheezes that were not responding to the treatment previously prescribed. She also was prescribed steroids as needed for relapses which happened frequently.

She sought help by multiple pulmonologists and internists, because it was interfering with her life activities, who prescribed her the same treatment and would put her on oral steroids which would improve the patient's condition partially but not completely.

Frequent exacerbations were noticed but since she was already taking the maximal optimal dose for asthma medications, nothing else could be done.

In our hospital, the patient was reevaluated with a detailed history focusing on the place of residence, degree of ventilation of her house (which was well ventilated) and exposure to allergens and animals at home. There was nothing of significant value with no allergen exposure, no history of NSAIDs or β-blockers use and no heart burn. She did however mention a brownish colored sputum with a plug like consistency and multiple courses of antibiotics.

She also mentioned that her husband is an avid smoker but not in front of her and her past medical and surgical history was unremarkable despite the asthma. She affirmed to be compliant with her medication but still suffering from frequent exacerbations that were partially relived by oral or IV steroids.

**Fig. 1:**
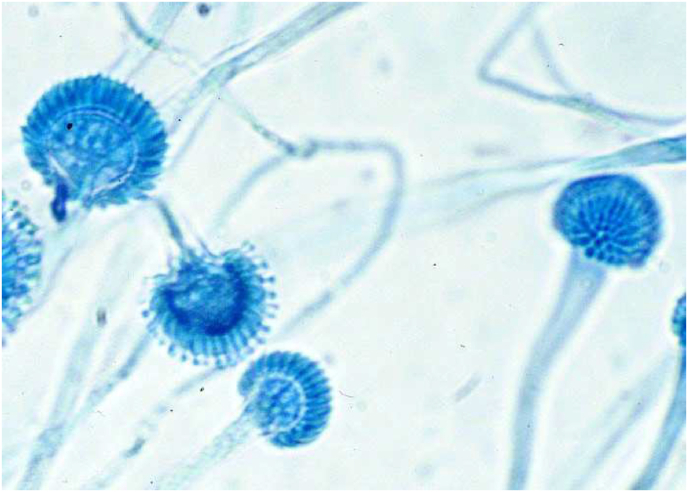
Hyphae seen on light microscope from the sputum culture.

This led to the suspicion of allergic bronchopulmonary aspergillosis and thus further investigations were done which included:

A chest-CT scan that showed atelectatic bands. Blood and sputum samples that showed increased IgE levels (450 kU/L), increased eosinophils count (1200 cells/mcL) and a sputum culture that revealed hyphae (Fig. 1).

Conclusively, the diagnosis of allergic bronchopulmonary aspergillosis was made, and the patient was started on itraconazole (400 mg x 1) and steroids (40 mg x 1).

After two weeks, on her follow up the patient showed significant improvement in her respiratory symptoms, thus her medications were tapered.

In a further follow up after two weeks, all her medications and nebulizers were stopped, and she was feeling well and free of symptoms.

In conclusion, after one month of our correct diagnosis and treatment, the patient was off medications and feeling well.

## 3 Discussion

ABPA is an immunologic pulmonary disease due to a hypersensitivity reaction to a fungus called *Aspergillus fumigatus*. Clinically, the patients that suffer from this disease present with chronic asthma, recurrent pulmonary infiltrates and bronchiectasis [1].

Because many of the patients tend to be asymptomatic or minimally symptomatic, there should be a high index of suspicion while managing any patient with bronchial asthma and that's why all patients with asthma should have routine screenings with an Aspergillus skin test [3,10].

The condition has a variable pathophysiology that is still largely unknown. Some papers define it as an immediate hypersensitivity (type 1) response, some as an antigen-antibody complexes (type 3) response and some as an eosinophil-rich inflammatory cell response (type 4b) [2,5].

The reason why asthmatic patients are susceptible to this disease is not fully understood, but some authors have reported that exposure to large concentrations of the *A fumigatus* spores may be the cause [12].

Environmental factors are not considered one of the main pathogenic factors because not all asthmatic patients develop ABPA even if they had been exposed to the same environmental factors [10].

The fungus persists and germinate into hyphae in a genetically predisposed individual, which causes the release of antigens that compromise the mucociliary clearance, stimulate and breach the airway epithelial barrier and activate the innate immunity of the lungs. This causes an influx from the inflammatory cells and a resultant early and late phase inflammatory reaction [10].

In recent years, it has been discovered that the fungus attaches to the lung epithelial cells resulting in the release of pro-inflammatory mediators and the influx of granulocytes which cause an intense Th2 CD4 T-cell immune response. The Th2 cytokines (interleukin [IL]-4, IL-5, and IL-13) lead to total and *A fumigatus*-specific IgE synthesis, mast cell degranulation, and promotion of a strong eosinophilic response [7,8].

A combination of these events can also be the cause for the development of progressive fibrosis and bronchiectasis. Although traditional treatment has focused on suppressing the immune reaction to these phenomena, with high-dose corticosteroids, the presence of viable organisms driving this process makes the option of antifungal chemotherapy an attractive adjunct [9,10].

There are five criteria that should be present to lead to the suspicion of ABPA in an asthmatic patient [3,5]:

• Asthma.
• Proximal bronchiectasis (dilated bronchi in the inner two thirds of the chest field on a CT-scan).
• Immediate cutaneous reactivity to *Aspergillus* species or *Aspergillus fumigatus.*
• A total serum IgE that is elevated (417 kU/L or 1000 ng/mL),
• Elevated serum IgE–*A fumigatus* and/or serum IgG-*A fumigatus.*

The signs and symptoms for this disease are very similar to those of asthma, which are: wheezes, shortness of breath and cough with brownish flecks or bloody mucous. It also can cause fever and weight lose but it is rare [5,7].

Control over symptoms of asthma and cystic fibrosis, prevention or treatment of pulmonary exacerbations, reduction or remission of pulmonary inflammation and reduction in the progression to end-stage fibrotic are the four goals of the treatment for ABPA, since a delay in the treatment can lead to complications such as pulmonary fibrosis, bronchiectasis, chronic sputum production and severe persistent asthma with loss of lung function [2].

In our Case, the patient had a clear history of asthma with shortness of breath and a brownish colored sputum production in addition to high serum IgE levels (as mentioned above) and hyphae from the sputum culture. All of which indicated an *Aspergillus* infection.

For the treatment of this condition, we can use systemic glucocorticoids and antifungal therapy such as itraconazole and voriconazole and omalizumab [2,6].

With early diagnosis, careful patient monitoring and appropriately aggressive treatment the prognosis of ABPA is generally favorable, but it is worse for patients who develop corticosteroid-dependent asthma or advanced stage fibrotic lung disease [6].

In our Case, it is clear the patient was in the process of developing a persistent asthma due to a late diagnosis. Fortunately, with a reevaluation of the patient's medical history, the signs and symptoms, the laboratory and histological findings we could correctly diagnose the patient's condition and successfully treat her which resulted in an optimal outcome.

The clue in this Case was the brownish sputum that led us to take ABPA into consideration as a differential diagnosis and do further investigations.

After we treated the patient with steroids and itraconazole, she improved to the point that she did not need medications or nebulizers. Thus, ultimately our patient had a good outcome, and we expect a good prognosis for her.

## 4 Conclusion

From this Case, we can conclude that it is very important to take a good medical history and physical exam because they are essential for a correct diagnosis. It is only due to the fact that we took a detailed history from the patient and her mentioning a brownish sputum production that we were led to take ABPA into consideration as a possible diagnosis.

In addition, if the commonly used medications for a disease do improve the patient's condition, then the patient should be reevaluated for another disease or a subjacent condition.

Fortunately, our diagnosis did not take long and thus our patient did not have to suffer from the chronic conditions that ABPA can lead to. Although, an early diagnosis is preferable for this condition and thus we should always keep ABPA in mind as a possible condition in asthmatic patients and patients with cystic fibrosis. That's why we suggest skin *Aspergillus* tests for patients that have asthma or cystic fibrosis especially in cases that do not improve with optimal treatment.

## Ethical approval

The study is exempt from ethical approval in our institution.

## Sources of funding

This research did not receive any specific grant from funding agencies in the public, commercial, or not-for-profit sectors.

## Authors’ contributions

Study concept or design: Mohammad Kharraz.

Writing the manuscript: Yousef S. Abuzneid, Yasmine Yaghi, Naser Amro, Arein Madia, Nataly Salhab, Sadi A. Abukhalaf.

Review & editing the manuscript: Yousef S. Abuzneid and Yasmine Yaghi.

## Registration of research studies

Not applicable.

## Guarantor

Dr. Yousef S. Abuzneid.

## Consent

Written informed consent was obtained from the patient for publication of this Case report and accompanying images. A copy of the written consent is available for review by the Editor-in-Chief of this journal on request.

## Declaration of competing interest

There is no conflict of interest.
